# Accuracy of Non-Enhanced CT in Detecting Early Ischemic Edema Using Frequency Selective Non-Linear Blending

**DOI:** 10.1371/journal.pone.0147378

**Published:** 2016-01-25

**Authors:** Georg Bier, Malte N. Bongers, Hendrik Ditt, Benjamin Bender, Ulrike Ernemann, Marius Horger

**Affiliations:** 1 Department of Diagnostic and Interventional Radiology, Eberhard Karls-University Tuebingen, D-72076, Tuebingen, Germany; 2 Siemens AG Healthcare, Imaging & Therapy Systems Computed Tomography & Radiation Oncology, Siemensstr. 1, D-91301, Forchheim, Germany; 3 Department of Neuroradiology, Eberhard Karls-University Tuebingen, D-72076, Tuebingen, Germany; INSERM U894, FRANCE

## Abstract

**Purpose:**

Ischemic brain edema is subtle and hard to detect by computed tomography within the first hours of stroke onset. We hypothesize that non-enhanced CT (NECT) post-processing with frequency-selective non-linear blending (“best contrast”/BC) increases its accuracy in detecting edema and irreversible tissue damage (infarction).

**Methods:**

We retrospectively analyzed the NECT scans of 76 consecutive patients with ischemic stroke (exclusively middle cerebral artery territory—MCA) before and after post-processing with BC both at baseline before reperfusion therapy and at follow-up (5.73±12.74 days after stroke onset) using the Alberta Stroke Program Early CT Score (ASPECTS). We assessed the differences in ASPECTS between unprocessed and post-processed images and calculated sensitivity, specificity, and predictive values of baseline NECT using follow-up CT serving as reference standard for brain infarction.

**Results:**

NECT detected brain tissue hypoattenuation in 35 of 76 patients (46.1%). This number increased to 71 patients (93.4%) after post-processing with BC. Follow-up NECT confirmed brain infarctions in 65 patients (85.5%; p = 0.012). Post-processing increased the sensitivity of NECT for brain infarction from 35/65 (54%) to 65/65 (100%), decreased its specificity from 11/11 (100%) to 7/11 (64%), its positive predictive value (PPV) from 35/35 (100%) to 65/69 (94%) and increased its accuracy 46/76 (61%) to 72/76 (95%).

**Conclusions:**

This post-hoc analysis suggests that post-processing of NECT with BC may increase its sensitivity for ischemic brain damage significantly.

## Introduction

Early treatment of ischemic stroke with an optimal time-window of 4.5h for therapeutic decision making is associated with an improved outcome [1]. Among all available imaging techniques, non-enhanced CT (NECT) is primarily most widely used, as it enables exclusion of brain hemorrhage as the main differential diagnosis to ischemic stroke and is quick to perform [2–6].

Ischemic stroke appears with different imaging characteristics on NECT, ranging from early ischemic changes based on ionic edema to infarcted, irreversibly damaged brain tissue. The morphological correlate for infarction and edema on NECT is commonly a tissue hypo-attenuation [7, 8], but the extent of early ischemic changes in NECT varies considerably and in many patients no abnormalities are found [9]. Also due to the narrow time window after onset of neurological deficits and since evidence from numerous studies has pointed to the predictive value of early ischemic changes seen on NECT for both functional outcome and the risk of hemorrhage, CT remains the workhorse in stroke imaging [10–13].

The purpose of our study was to assess whether post-processing by a new, frequency-selective non-linear blending algorithm called “best contrast” (BC) could increase the diagnostic sensitivity for detection of brain tissue changes associated with brain infarction.

## Materials and Methods

The ethics committee of the Eberhard Karls University Tuebingen approved this retrospective study with a waiver for the need of informed consent. Patient records were de-identified and analyzed anonymously. The study included consecutive patients with sudden onset of neurological deficits suggesting acute stroke in the MCA territory. According to a recent study by Fischer et al., patients were subclassified in minor and major stroke (threshold NIHSS value of ≤3) [14]. All patients were admitted to the stroke unit and underwent CT-imaging first, according to the in-house management of stroke patients and all patients underwent subsequently systemic thrombolysis. Follow-up NECT imaging studies evaluated the course of the ischemic stroke.

### CT Scanning protocol

All imaging was conducted on a 16-section multidetector scanner (Sensation 16 CT; Siemens Medical Systems, Forchheim, Germany).

First NECT was performed from the skull base to the vertex using the following imaging parameters: 120 kVp, 285 mAs, collimation 0.75mm, FOV = 220mm, 4.5mm slice thickness, matrix 512x512, soft tissue kernel (H30s).

Subsequently, CT-perfusion (CTP) was performed for confirmation of diagnosis and therapy decision making, but the data was not further analysed in this study.

Additionally, a *CT-angiography* (CTA) covering the area from the carotid bifurcation to the vertex was performed. It was done as follows: 0.7 mL/kg contrast, 5- to 10s delay from injection to scanning using 120 kVp, 366 mAs, 0.75 mm collimation, 200 mm FOV, H20f kernel with 0.7 and 1mm reconstructed slice thicknesses and coronal and sagittal MPRs.

### Post-imaging therapy

Patients within the time-window of <4.5h after onset of symptoms with either early signs of ischemia, CBF/CBV-mismatch or mismatch between the area of perfusion deficit and hypoattenuated ischemic areas in non-enhanced CCT were treated by either systemic thrombolytic therapy and/or endovascular thrombectomy: 2 patients (2.6%) received systemic thrombolytic therapy only, 3 patients (3.9%) underwent intravascular recanalization without additional intra-arterial thrombolysis and 71 patients (93.5%) underwent combined thrombolytic therapy and intra-arterial thrombectomy. The success of therapy was evaluated using the thrombolysis in cerebral infarction (TICI) scale [15].

We will not go into detail regarding stroke therapy as this was not the focus of this study.

### Image analysis

For standard *NECT*-reading two different window settings 80HU width and 40HU level and 35-45HU width and 35-45HU level were used. Two readers (M.H. and G.B.) with 20 and 4 years of experience in reading brain CT, who were blinded to the clinical diagnosis, the symptoms and the results of the CTP at the time of interpretation, performed the image analysis. They both assessed the localization and extent of ischemic brain edema represented by tissue hypo-attenuation in the MCA-territory in two image slabs: The top edge of the upper slab was just above the level of the lateral ventricle (supraganglionic level), and the bottom edge of the lower slab was at the level of the basal ganglia (ganglionic level). The extent of hypo-attenuating tissue was measured manually in each slice by drawing a freehand ROI. The localization of hypo-attenuated tissue changes were classified according to the Alberta stroke program early CT score (ASPECTS) [16]. The MCA territory was divided into 10 regions of interest on two transversal CT slices. The regions are as follows: caudate nucleus, insula, lenticular nucleus, internal capsule and six cortical regions designated M1-M6. The areas and the locations of hypo-attenuated areas in the MCA-territory were hand drawn, both on NECT and follow-up NECT at both ganglionic- and supraganglionic level (2 CT slices). The follow-up NECT was performed following the same CT-protocol, serving as reference standard. Hemorrhages which occurred on follow-up NECT were classified according to the Heidelberg classification [17].

### Best contrast image post processing

**Fig 1:**
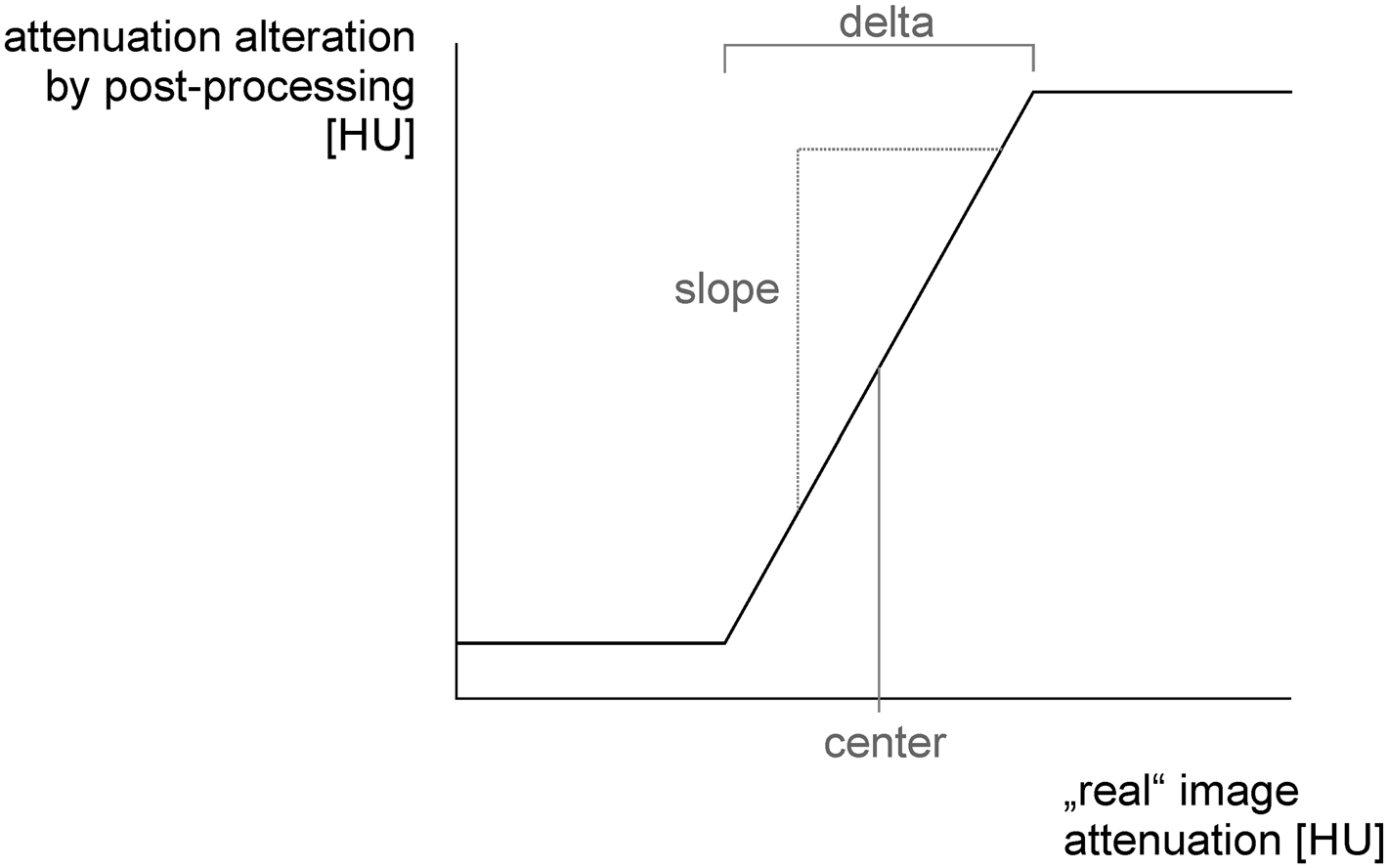
Schematic illustration of the “best contrast” (BC) post-processing. The center is defining the attenuation level, at which image information is highlighted. By adjustment of the slope and delta of the attenuation curve, the expression of the post-processing can be modulated.

BC-images were computed using a new algorithm enabling non-linear blending in image space, implemented in a client server application of the manuracturer (SyngoVia, Research Frontier, Siemens Healthcare, Germany). The algorithm first divides the original image information into low and high frequencies, whereas high frequencies represent the main part of image noise and low frequencies include contrast information after discrete Fourier transformation. The thresholds for the applied high- and low-pass-filters depend on the maximum and minimum frequencies of the original image information and are processed after Fourier transformation. Second, a non-linear scaling function is propagated to low frequencies to highlight pixel intensities in a defined subset of the entire dynamic range (delta). Intensity of highlighting can be influenced by adapting the slope and delta of the subset (Fig 1). At last, high frequencies of the original image are merged with adapted low frequencies to BC images with augmented contrast values. The best settings of tissue contrast differentiation, using this new algorithm, was set at a center of 30 HU and a delta of 5 HU at a slope of 5. Settings of each data set were recorded and all image sets were computed using the averaged settings of both readers.

To exclude potentially false positive results in the initial BC image reading, follow-up NECT images were also transferred into BC images and evaluated (in terms of ASPECTS grading) separately in randomized order by both readers without knowledge of the results of the admission CT reading.

### Statistical analysis

Statistical analyses were performed using JMP 11.2 statistical software (SAS Institute Corp., Cary, North Carolina, USA). The Kolmogorow-Smirnov test was performed for normality testing. In cases with normal distributed data (extent of hypoattenuated area) paired student’s t-tests were calculated for comparison.

Assessment of diagnostic accuracy in terms of sensitivity, specificity, positive and negative predictive value was performed using follow-up-NECT as reference standard.

For comparison of the detection rate of hypoattenuated brain areas Fisher’s exact test was applied. For comparison of the different ASPECT scores (NECT vs. BC) Bland & Altman statistics were performed.

Nominal data as the influence of the localization of the vessel occlusion and clinical data on the stroke area (TIA, minor/major stroke) were assessed by linear regression analysis.

Inter-observer agreement was assessed by Cohen’s kappa (k).

For all tests, p values < 0.05 were considered statistically significant. 95%-confidence intervals are given if not indicated otherwise.

## Results

### Patient characteristics and clinical data

A total number of 76 patients who underwent NECT for stroke diagnosis at our institution between 02/2011 and 07/2015 were included in this retrospective analysis. Based on the clinical examination 2 patients (2.6%) were classified as having had a minor stroke (NIHSS ≤ 3) and 74 patients (97.4%) as having had a major stroke. In this study, no patient with a transitory ischemic attack (TIA) was included. The median National Instituted of Health stroke scale (NIHSS) value was 13 (range: 3–25; 95%-CI: 11–14), but only available in 53 cases due to the retrospective study design. Based on imaging data including NECT, CTA and CTP, the localization of the stroke was right-sided in 39 cases (51.3%) and left-sided in 37 cases (48.7%). The localizations of the causal vessel occlusion/stenosis are displayed in Table 1. The results of intravascular therapy were considered to be successful in 68 cases (91.8% of all recanalization attempts) with nearly complete or complete perfusion of the affected arterial territory (TICI grade 2b/3).

**Table 1:** Patients’ demographics.

No. of patients	76	%
**Age, years, mean ± SD**	68.42 ± 11.99	
Age range	(29–90)	
**Gender**		
Male	49	64.5
Female	27	35.5
**Localization of vessel occlusion**		
ICA (isolated)	22	29
M1 (isolated)	31	40.8
M2 (isolated)	1	1.3
M1+M2	2	2.6
ICA+M1	18	23.7
ICA+M2	1	1.3
ICA+M1+M2	1	1.3

### NECT results

NECT image analysis yielded 35 patients with hypodensities and an ASPECTS score of 10 (no measurable hypo-attenuating area) in 41 patients (53.0%). An ASPECTS score of 9 was given in 24 patients (32.6%), 8 in 6 patients (7.9%), 7 in 3 patients (3.9%), and a score of 6 and 4 was given in 1 patient each (1.3%).

Non-enhanced brain CT yielded a total of 55 hypoattenuated brain lesions with a high inter-observer agreement (k = 0.81), predominantly distributed in the lentiform nucleus (n = 22; 28.9%), followed by the insular ribbon (n = 8; 10.5%) and the M1, M2 and M4 cortex regions (each n = 5; 6.6%). The caudate nucleus (n = 3; 3.9%), internal capsule (n = 1; 1.3%), M3 (n = 2; 2.6%), M5 (n = 3; 3.9%) and M6 cortex region (n = 1; 1.3%) presented lower numbers of hypoattenuated lesions on NECT. The mean infarct area measured in NECT was 7.9±7.7cm^2^ (95%-CI: 5.98–10.01cm^2^; range: 1.08–32.7 cm^2^) with substantial inter-observer-agreement (k = 0.69).

### BC results

The ASPECTS score after BC image analysis was defined as 10 in 5 cases (6.6%), 9 in 32 cases (42.1%), 8 in 24 cases (31.6%), 7 in 9 cases (11.8%), 6 in 3 cases (3.9%), 4 in 2 cases (2.6%) and 2 in 1 case (1.3%).

**Fig 2:**
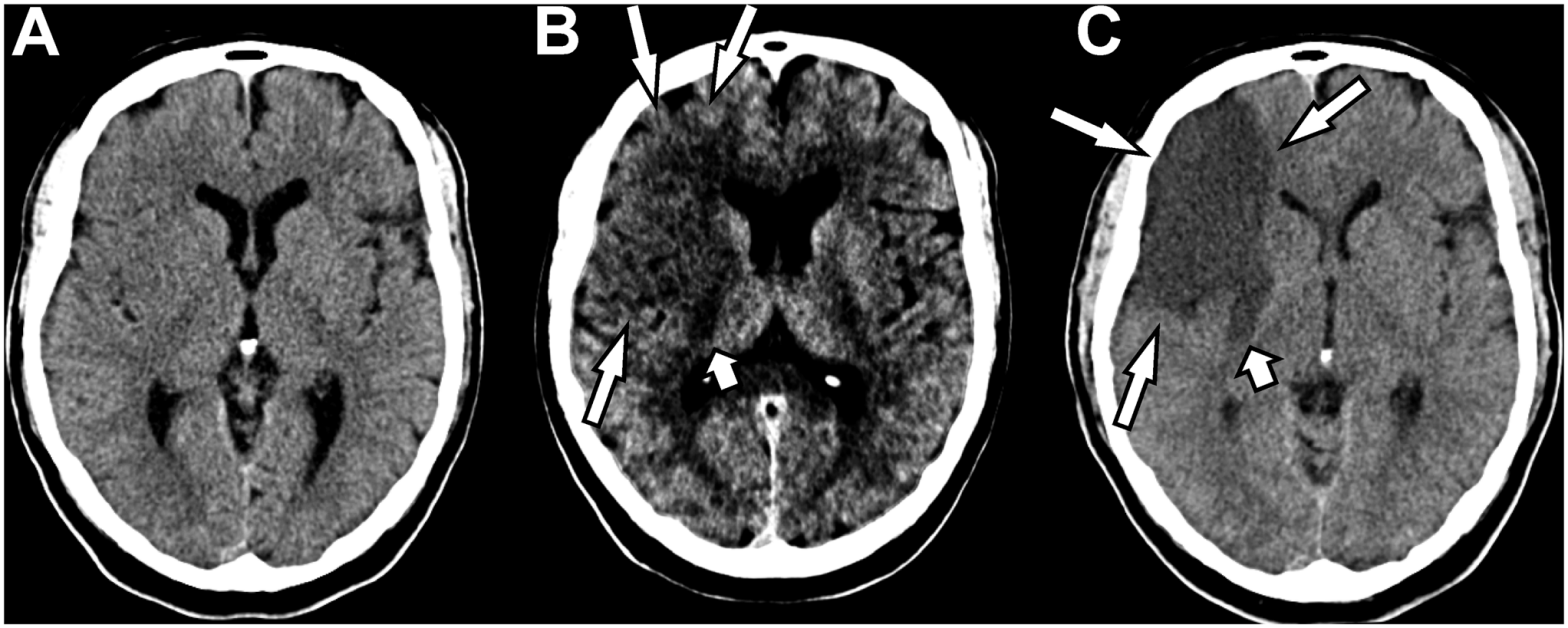
64 year-old male patient with ischemic stroke of the right cerebral hemisphere due to an isolated occlusion of the right internal carotid artery. While the NECT (A) only give an idea of a slight dedifferentiation of gray and white matter at the insular cortex, the “best contrast” image (B) shows a great delineated infarction (long arrows) with involvement of the internal capsule (short arrow). The diagnosis was confirmed on follow-up CT, which shows that the BC image reflects the infarct size more accurately (C).

A total number of 139 hypoattenuated brain lesions were described (inter-observer agreement: k = 0.76), most of them localized in the lentiform nucleus (n = 41; 53.9%), followed by the insular cortex (n = 27; 35.5%) and the M2 cortex area (n = 18; 23.7%). Moreover, 11 lesions (14.5%) were found in the M5 region, 10 each (13.2%) in the caudate nucleus, internal capsule and M4 region, 6 (7.9%) in the M1 cortex area, 4 (5.3%) in the M4 cortex area and 2 (2.6%) in the M6 cortex area. An example of a patient is given in Fig 2.

**Fig 3:**
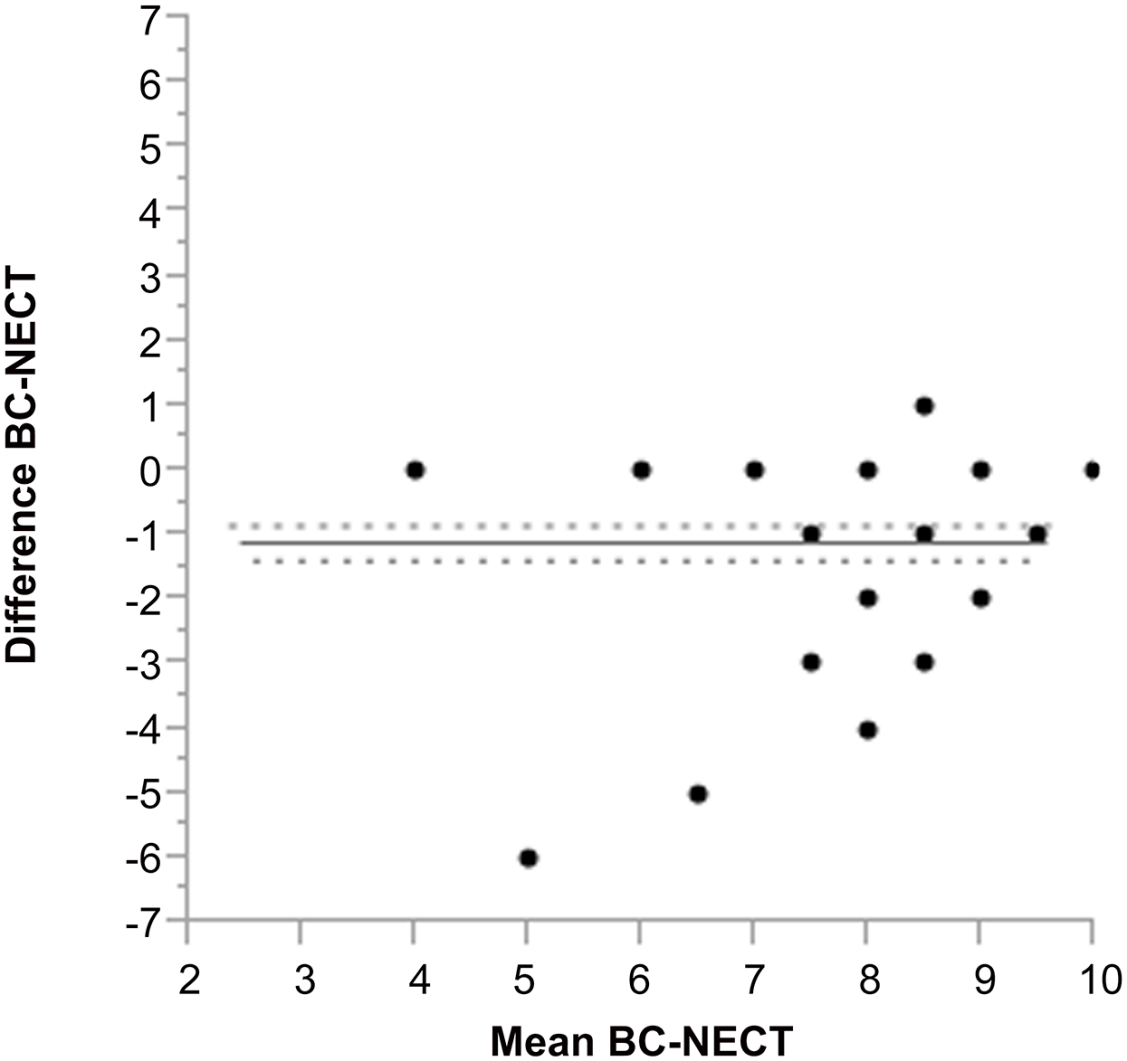
Bland & Altman plot of the ASPECTS score of the NECT analysis in comparison to the “best contrast” image analysis.

The mean ASPECTS difference between BC and NECT analysis was 1.1 (95%-CI: 0.84–1.37) and statistically different (p<0.0001; Fig 3). The pairwise correlation between the mean value BC-NECT and its difference was 0.14 (Spearman’s τ and Kendall’s tau). In a region-specific Bland & Altman analysis, significant differences were found regarding the detection rate in the caudate nucleus (p = 0.0073), the insular cortex (p<0.001), the internal capsule (p = 0.002), the lentiform nucleus (p<0.001), the M2-region (p = 0.002), the M4-region (p = 0.024), M4-region (p = 0.004) but not in the M1-region (p = 0.321), the M3-region (p = 0.321) and the M6-region (p = 0.321).

The mean area of hypo-attenuated brain areas in BC-images was found to be 8.12±7.59cm^2^ (95%-CI: 6.32–9.91cm^2^; range: 1.34–37.5cm^2^) with substantial inter-observer-agreement (k = 0.64).

Regarding the hypo-attenuated area measurements in the BC and NECT images a mean difference of 3.08 (95%-CI: 0.52–5.63 cm^2^) was measured (p = 0.019).

### Outcome analysis

**Fig 4:**
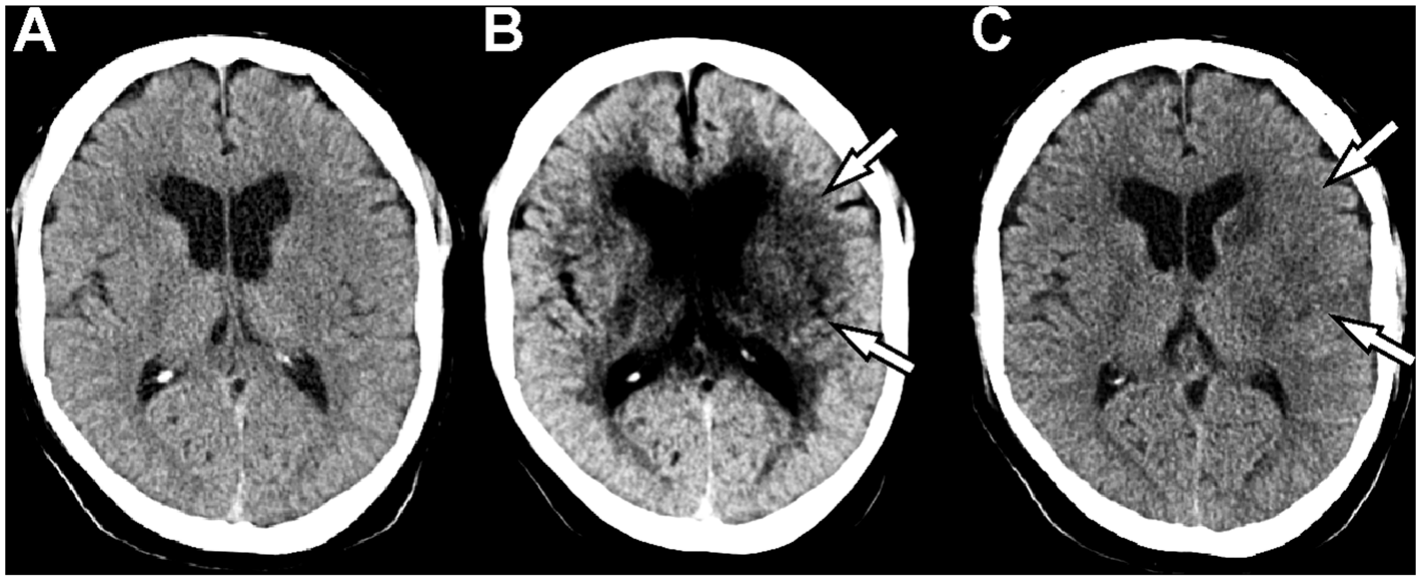
50-year-old male patient with ischemic stroke of the left hemisphere due to occlusion of the left internal carotid artery (ICA). The NECT (A) does not show a significant infarct demarcation, whilst the “best contrast” optimized images (B) show a demarcation of the left basal ganglia and the insular cortex (arrows), which was confirmed by follow-up CT (C).

Follow-up CT was conducted in every patient within 5.73±12.74 days after admission NECT. In 65 patients (85.5%) hypoattenuated lesions were described as correlate for demarked infarction. Of these patients n = 35 (53.8%) were correctly identified by the initial NECT and all (65; 100%) by the “best contrast” image analysis (Fig 4), yielding a sensitivity of 54% (95%-CI: 41.1–66.1%) for NECT and 100% (95%-CI: 93.0–100%) for the BC image analysis. The specificity was 100% for the NECT analysis (11/11 true negatives; 95%-CI: 65.5–100%) and 64% for the BC image analysis (7/11 true negatives; 30.8–89.1%). Negative and positive predictive value (NPV/PPV) as well as accuracy were calculated and are tabulated in Table 2. The difference between both methods was statistically significant (p = 0.012).

**Table 2:** Patient based diagnostic accuracy of NECT vs. BC.

Method	Sensitivity	Specificity	PPV	NPV	Accuracy
**NECT**	54%	100%	100%	27%	61%
**BC**	100%	64%	94%	100%	95%

In a lesion-based approach the sensitivity of NECT for detection of hypo-attenuated lesions was 34% (95%-CI: 24.9–44%) vs. 83.3% (95%-CI: 73.6–89.8%) for the BC image analysis. Lesion-based, the difference between both methods showed to be significant, too (p<0.05).

Regarding the ASPECTS score at FU, a 10 was reached in 11 cases (14.5%), 9 in 21 cases (27.6%), 8 in 19 cases (25%), 7 in 6 cases (7.9%), 6 in 7 cases (9.2%), 5 in 2 cases (2.6%), 4 in 3 cases (3.9%), 3 in 1 case (1.3%), 2 in 5 cases (6.6%) and 1 in 1 case (1.3%). The recanalization attempts were unsuccessful in 6 cases (8.2%) and post-interventional hemorrhage was detected in 12 patients (15.8%). The extent of infarction in these patients may-have been significantly increased and these patients were therefore excluded from further analysis.

The extent of hypoattenuated areas correlated moderately for the NECT analysis (r = 0.683; p = 0.138). However, significance was not given due to the reduced patient number with detectable and measurable lesions. The areas measured after BC image analysis yielded a good correlation with the final outcome (FU-NECT) area (r = 0.802; p<0.0001).

In a lesion-based analysis, 58 finally demarked lesions (30.4%) were not detected by NECT analysis, whilst the BC-image reading yielded a number of 15 undetected lesions (7.9%). The specific localizations of the non-detected lesions are displayed in Table 3. The size median of these missed lesions was 6.92 cm^2^ for the NECT reading (range: 1.3–19.21 cm^2^) and 7.2 cm^2^ for the BC-image analysis (range: 4.6–13.8 cm^2^). The differences in size were not considered statistically significant (p>0.05).

**Table 3:** Initially not detectable hypo-attenuated brain parenchymal areas.

Region	NECT	Best Contrast
**Caudate nucleus**	3 (5.2%)	0 (0%)
**Insular cortex**	10 (17.2%)	1 (6.7%)
**Internal capsule**	5 (8.6%)	1 (6.7%)
**Lentiform nucleus**	13 (22.4%)	0 (0%)
**M1**	1 (1.7%)	2 (13.3%)
**M2**	11 (19.0%)	2 (13.3%)
**M3**	3 (5.2%)	3 (20%)
**M4**	6 (10.4%)	1 (6.7%)
**M5**	5 (8.6%)	4 (26.6%)
**M6**	1 (1.7%)	1 (6.7%)
**Total**	**58**	**15**

Number and percentage of undetected hypo-attenuated areas in the initial non-enhanced CT and “best contrast” image analysis by a lesion-by-lesion evaluation.

*Abbreviations*: M1-M6 (MCA regions defined according to Alberta early CT score (ASPECTS)

Post-interventional hemorrhage was detected in 12 cases (15.8%). According to the Heidelberg bleeding classification [17] a hemorrhagic infarction (HI) type HI1 occurred in 2 cases (16.7%), type HI2 in 3 cases (25%), a parenchymal hematoma (PH) type PH1 in 1 case (8.3%) and PH2 in 6 cases (50%).

## Discussion

The detection of ischemic edema and early brain tissue damage by NECT is usually difficult in particular within the therapeutic window. Our study shows that using non-linear blending based post-processing increases the sensitivity of NECT for detection of ischemic edema from 54% to 100% with decreasing specificity from 100% to 64% in this specific patient cohort.

In comparison to this a sensitivity of 57% and specificity of up to 100% for acute stroke detection on non-enhanced CT with potential for further increase up to 71% by using modified window settings (window width of 8HU and center level of 32HU), were reported in the specialty literature [18]. With another approach, using contrast-enhanced CT, delineation between ischemic areas and normal brain parenchyma can be made slightly more visible. In a previous work [19] the maximum parenchymal enhancement of the cortex, white matter and the ganglia was achieved 30-40s after bolus injection. These authors demonstrated different magnitudes of increase in attenuation in the cortex (9.4HU) and basal ganglia (8.2HU) versus white matter (4.6HU) between non-enhanced and enhanced series with the effect of an increased tissue contrast e.g. at the corticomedullary junction and between ischemic and normal brain tissue. A step towards quantification of ischemia was proposed by using a standardized assessment of ischemic changes according to the so-called Alberta Stroke Program Early CT Score (ASPECTS) [20].

Complementary, other CT-examinational techniques like CT angiography (CTA) and/or perfusion-CT have been implemented over the course of the last few years, in particular in specialised centers (stroke units) with the purpose of a better management of stroke patients. CTA is used in order to localize thrombi within intracranial vessels and for evaluation of collateral circulation. Especially the extent of collateralization has become an important parameter for the early estimation of tissue at risk and potentially salvageable brain regions [21]. Thus, it is a more and more valuable tool for clinical decision making for endovascular therapy. Notably, in an earlier report, NECT, CTA and CTP as well as MRI DWI were found to not significantly differ with regard to prediction of disability at 90 days following minor stroke or TIA [22]. According to previously published data, an area of parenchymal hypo-attenuation documented on NECT scans has a low probability of decreasing in size or of becoming normal after thrombolytic therapy [19].

The use of BC reading improved not only detection of ischemic lesions but also more precisely delineated the extent of early ischemia with a mean involved area of 7.7±7.4cm^2^ for NECT and 8.12±7.5cm^2^ for BC. Hypo-attenuating brain parenchyma as correlate for ischemic brain tissue with ionic edema reflects irreversibly damaged tissue and is therefore considered a potential risk factor for post-interventional hemorrhage and accurate depiction of the infarction is therefore relevant for planning thrombolytic therapy. Due to the more noticeable contrast between normal brain parenchyma and ischemic areas, the inter-observer agreement proved superior for BC-reading as well.

A persistent hypo-attenuated area at FU-NECT as a correlate for demarked infarction was detected in 65 patients (85.5%) on follow-up as correlate for irreversible brain parenchyma damage. Of these, 47.2% were missed by the initial standard NECT reading whereas all (100%) were correctly identified by BC image analysis (p = 0.012). In particular brain ischemia in the insular cortex, lentiform nucleus as well as in part in the cortical brain areas was more frequently underdiagnosed by standard NECT-reading. However, a lesion-by-lesion evaluation, disclosed some overseen hypo-attenuated areas also for BC reading. They were almost all located in the cortical brain areas which are prone to more beam hardening artifacts caused by the thick cranial vault bones lying next to the brain cortex and are thus more susceptible to false negative results.

Our study has some limitations. First, it was evaluated retrospectively and therefore clinical information is reduced, so further prospective observations are needed. Second, follow-up is not the ideal reference standard and the initial extent of brain parenchyma abnormality and the discrimination between irreversible brain-damage and edema is not possible. Especially in cases with secondary hemorrhage, the extent can be increased on FU-NECT. Third, only the areas of maximum hypo-attenuation were measured, therefore bias due to different patient positioning may occur between NECT and FU-CT. Fourth, with a median NIHSS of 13 the included patient cohort is moderately severe affected. Moreover, only patients that received thrombectomy or thrombolysis, are included in the study. Therefore, the applicability to all stroke patients and the generalisability of this study is limited.

Nevertheless this study shows that a significant improvement in image quality and subsequently diagnostic accuracy is possible in stroke patients undergoing NECT by using a frequency selective non-linear blending algorithm. However, the meaning of hypo-attenuating brain parenchyma as well as the value of the ASPECTS score on admission CT for therapy planning is still point of discussion [23, 24] and prospective studies are needed to further evaluate our preliminary results and their potential influence on therapy and follow-up imaging.

## Supporting Information

S1 Table Patients data. Basic data of the patients collective.
(XLSX)
